# Liquefied Wood as Inexpensive Precursor-Feedstock for Bio-Mediated Incorporation of (*R*)-3-Hydroxyvalerate into Polyhydroxyalkanoates

**DOI:** 10.3390/ma8095321

**Published:** 2015-09-23

**Authors:** Martin Koller, Miguel Miranda de Sousa Dias, Alejandra Rodríguez-Contreras, Matjaž Kunaver, Ema Žagar, Andrej Kržan, Gerhart Braunegg

**Affiliations:** 1 Institute of Chemistry, University of Graz, NAWI Graz, Heinrichstrasse 28/III, Graz 8010, Austria; 2 ARENA—Association for resource efficient and sustainable technologies, Inffeldgasse 23, Graz 8010, Austria; E-Mail: g.braunegg@tugraz.at; 3 Department of Genetics, Institut de la Vision, 17 rue Moreau, 75012 Paris, France; E-Mail: miguel.dias@inserm.fr; 4 Departament d’Òptica i Optometria, Universitat Politècnica de Catalunya Barcelona Tech, Sant Nebridi 22, 08222 Terrassa, Barcelona, Spain; E-Mail: sandra8855@hotmail.com; 5 Laboratory for Polymer Chemistry and Technology, National Institute of Chemistry, Hajdrihova 19, 1000 Ljubljana, Slovenia; E-Mails: matjaz.kunaver@ki.si (M.K.); ema.zagar@ki.si (E.Z.); andrej.krzan@ki.si (A.K.)

**Keywords:** biopolymers, copolyester, (*R*)-3-Hydroxyvalerate (3HV), liquefied wood, polyhydroxyalkanoates (PHA), precursor substrates

## Abstract

Liquefied wood (LW) prepared in a microwave process was applied as a novel; inexpensive precursor feedstock for incorporation of (*R*)-3-hydroxyvalerate (3HV) into polyhydroxyalkanoate (PHA) biopolyesters in order to improve the biopolyester’s material quality; *Cupriavidus necator* was applied as microbial production strain. For proof of concept, pre-experiments were carried out on a shake flask scale using different mixtures of glucose and LW as carbon source. The results indicate that LW definitely acts as a 3HV precursor, but, at the same time, displays toxic effects on *C. necator* at concentrations exceeding 10 g/L. Based on these findings, PHA biosynthesis under controlled conditions was performed using a fed-batch feeding regime on a bioreactor scale. As major outcome, a poly(3HB-*co*-0.8%-3HV) copolyester was obtained displaying a desired high molar mass of *M_w_* = 5.39 × 10^5^ g/mol at low molar-mass dispersity (*Đ_M_* of 1.53), a degree of crystallinity (*X_c_*) of 62.1%, and melting temperature *T_m_* (176.3 °C) slightly lower than values reported for poly([*R*]-3-hydroxybutyrate) (PHB) homopolyester produced by *C. necator*; thus, the produced biopolyester is expected to be more suitable for polymer processing purposes.

## 1. Introduction

Nowadays, the biopolymer market is emerging extremely fast; in 2010, the global value reached a magnitude of US-$ 10^10^ with an obvious upwards trend. From 2006 to 2012, the quantity of global bioplastic production rocketed from 186 to 545 kt, about 90% thereof is dedicated to various packaging materials [1]. In this context, it has to be stressed that various polymer-based products are merchandized, which are tagged by the manufactures with the stylish and relatively easily marketable attribute “*green plastic*”. Often, the *de facto* properties of these commercialized materials do not really match the strict definitions as prescribed for classifying them as “*biobased*”, “*biodegradable*”, “*compostable*” or “*biocompatible*”. These attributes only apply to plastics fulfilling strict requirements as defined by standardized norms [2]. Polyhydroxyalkanoates (PHAs), natural polyesters produced by microbes from renewable resources, entirely fulfill these requirements [3]. The spectrum of potential applications for PHAs ranges from simple packaging materials featuring advantageous properties such as a high oxygen barrier and UV-resistance, to high-quality materials to be used in special niches, e.g., in the medical and pharmaceutical field, as thermo-sensitive adhesives, or as “smart latexes” [4]. Up to now, economic reasons have been pointed out as the major obstacle for replacing common plastics by PHAs on a relevant scale [3,4,5,6,7].

In nature, PHAs fulfill important biological tasks, mainly as energy and carbon reserve materials for the microbial cells [7]. Under conditions of lacking extracellular carbon sources, they can be remobilized and utilized as carbon and energy substrates. PHAs are key-compounds for the regulation of intracellular energy flow, e.g., for cell motility or osmoregulation, and contribute to distribution and routing of carbon reserves to different metabolic pathways. In addition, several important functions of PHAs in various ecosystems were elucidated, such as protection against environmental stress conditions such as osmotic shock, UV irradiation, desiccation, or thermal and oxidative stress [7]; in addition, the role of PHA for maintenance of the intracellular redox state was discussed [8]. Further, microbial PHA synthesis and degradation plays a crucial role for syntrophic interactions between consortia of different organisms in aquatic or terrestrial ecosystems [7]. In general, PHA accumulation is provoked by an availability of carbon source exceeding the strain’s requirements to master the demand for maintenance energy together with restricted supply with macro-components like nitrogen, phosphate or dissolved oxygen, or micro-components like magnesium, sulfate, and certain (heavy) metals [7,9]. After their biosynthesis, PHAs can be processed directly for generation of plastic-like bio-materials [10] or, together with various compatible organic or inorganic materials, they can be used to generate novel blends and composites with advanced properties [11,12,13], or they can be converted to functional follow-up compounds [14].

As a function of various factors like the microbial production strain, the nutrient supply regime, and the cultivation conditions during biosynthesis, PHA polymer chains, which are organized in granules, contain a magnitude of 10^2^ to 10^5^ 3-hydroxyalkanoic acid (3HA) monomers. In most cases, 3HAs are enantio-pure, *R*-configured chiral compounds. Among all known PHAs, poly[(*R*)-3-hydroxybutyrate] (PHB), the homopolyester of (*R*)-3-hydroxybutyrate (3HB), is the best characterized representative. PHB features rather high crystallinity, brittleness, and therefore restricted processability. The low difference between the decomposition temperature (around 270 °C) and the high melting point (around 180 °C) displays a too narrow “window of processability” for many processing techniques, e.g., in melt extrusion or production of polymeric films. These drawbacks can be overcome by interrupting the crystalline PHB matrix by additional building blocks like (*R*)-3-hydroxyvalerate (3HV) or the achiral building block 4-hydroxybutyrate (4HB), resulting in copolyesters with enhanced material properties and a broader range of applications [15]. The exact material properties are strongly dependent on the monomeric composition of the copolyesters, which can be triggered during microbial PHA biosynthesis by providing structurally related precursor substrates to obtain the desired building blocks [16]. For example, 3HV building blocks are produced by many PHA accumulating strains by supplying precursors structurally related to 3HV, namely odd-numbered fatty acids such as propanoic acid, pentanoic acid or heptanoic acid [17]. Unfortunately, the expenses for these 3HV precursors contribute significantly to the entire PHA production costs [7,18]. Hence, cheap alternative 3HV precursors are required, e.g., such obtained by ozonolytic degradation of odd-numbered fatty acids [17].

After adequate upstream processing, various renewable materials display the potential to act as feedstocks for the generation of such 3HV precursors. Primary for suitability is the substrates’ ability to be transformed into the desired monomer, which is dependent on the strain used, however abundance at (very) low cost is a non-negotiable necessity. Lignocellusosics coupled with an efficient treatment is an obvious choice for exploration. Wood is an abundant lignocellulosic raw material available in vast quantities; nevertheless, its natural high robustness makes is hardly accessible for direct bio-conversion towards value-added products. Kržan and Žagar [19] showed the efficiency of using untreated wood coupled with a microwave-assisted technique. The process was applied to acid-catalyzed hydrolysis of wood residues from forestry by using glycols and *p*-toluenesulfonic acid as acidic catalyst. By rapid heating to temperatures of about 200 °C, complete liquefaction was achieved within 7 min. The microwave-assisted liquefaction results in a “cocktail” of degradation products (liquefied wood, LW) displaying a source of odd-numbered compounds such as 4-oxopentanoic acid (levulinic acid) [20,21]. These compounds are potential substrates supporting 3HV biosynthesis by selected microbial production strains like *Burkholderia cepacia* [22], *Alcaligenes* sp. SH-69 [23], genetically modified *Pseudomonas putida* [24], or, as also used for the study at hands, *Cupriavidus necator* [25,26]). *C. necator* was selected due to the outstanding volumetric PHA productivity of this strain, its high potential to incorporate 3HV into PHA, and its high genetic stability [25,26].

The study was dedicated to get in insight into the possibility to implement LW as a precursor for *C. necator*-mediated PHA-copolyester biosynthesis.

It has to be considered that the chemical reaction mechanisms that drive the wood liquefaction process are not entirely elucidated in all details. Various low molar mass compounds and hemicelluloses are directly dissolved, while polysaccharides and lignin undergo catalyzed degradation reactions yielding various products such as levulinic acid. It has been shown that polysaccharides are first converted to glycosides, which subsequently are hydrolyzed to levulinic glycolides [19].

## 2. Results

### 2.1. Shake Flask Experiment

**Figure 1:**
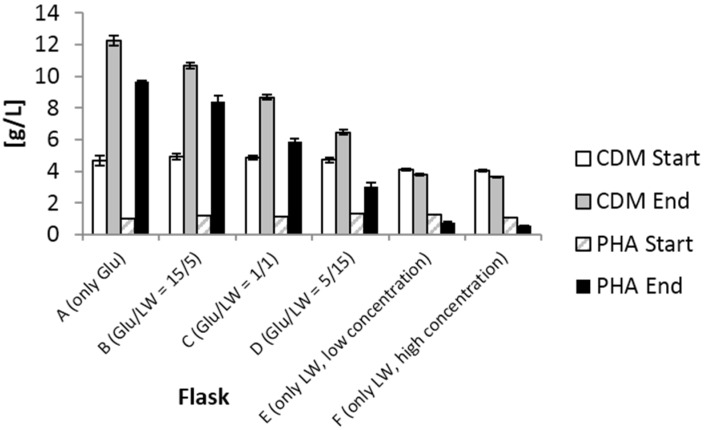
CDM and PHA concentration under PHA-accumulating conditions at the start (0 h) and at the end (24 h) of the shake flask cultivations. Data presented refer to mean values of two parallel cultivation; error bars reflect standard deviations of two parallel set-ups.

Figure 1 shows the concentrations of cell dry mass (CDM) and PHA both at the start and at the end of the shake flask experiments. Here, it is very visible that, using higher LW concentrations (set-ups E and F; LW as only carbon source), PHA concentration was lower at the end of the accumulation phase than at its beginning. This is due to the inhibiting effects of LW-constituents in these concentration ranges; similar findings for application of bagasse hydrolysates without prior treatment for removal of inhibiting components (formic acid, acetic acid, furfural, *etc.*) were reported previously [25]. It is assumed that the microbial cells consumed the PHA already accumulated during the growth phase instead of converting extracellular carbon source for PHA biosynthesis. In the case of the set-ups, A (glucose as only carbon source, no addition of LW), B (ratio glucose/LW = 15/5), and C (ratio glucose/LW = 10/10), a significant increase in PHA concentration was observed. Starting from a concentration of 1 g/L, final concentrations of about 9 (A), 8 (B), and 6 (C) g/L were measured. Regarding the set-ups D (ratio glucose/LW = 5/15), PHA concentration increased to about 3 g/L. Data for the intracellular mass fraction of PHA in CDM were calculated as follows (g PHA/g CDM): 78.8 (set-ups A), 78.7 (set-ups B), 68.0 (set-ups C), 46.6 (set-ups D), 20.0 (set-ups E), and 14.5 (set-ups F). Yields for PHA production from carbon sources amounted to (g/g): 0.43 (set-ups A), 0.36 (set-ups B), 0.24 (set-ups C), 0.08 (set-ups D); in set-ups E and F, the final values for PHA were lower than at the beginning. Volumetric productivities were calculated as follows (g PHA/L h): 0.36 (A), 0.30 (B), 0.20 (C), 0.07 (D) (no valued for set-ups E and F, because lower final PHA concentration than start concentration).

Table 1 shows the data for polymer composition, molar mass distribution (weight average molar mass *M_w_*, number average molar mass *M_n_*, polydispersity *Đ_M_*) and thermal characterization (*T_m_*, *T_g_*, *T_c_*) of the obtained polyesters. As expected, the highest molar masses and highest melting temperatures were displayed by the homopolyester PHB (set-ups “A”). Melting points decreased in parallel with the slight increase of the 3HV content in the polymers isolated from the setups B and C as determined via GC-FID. This is also true for setups D and E, where, due to the inhibiting effects of LW, no polymer accumulation took place during the phase of nitrogen limitation. In these cases, degradation of the polymer has obviously taken place, resulting in lower melting points, lower molar masses and slightly increased dispersities (*Đ_M_*). It has to be emphasized that all *Đ_M_* values are quite low and indicate a narrow distribution of molar masses. Hence, the polyesters exhibit a highly uniform length of the intracellular polymer chains. Polymer production in set-ups F was too low to allow PHA recovery for characterization.

**Table 1:** Polymer data of the different shake flask set-ups.

Set-up	Carbon Feed (GLU/LW)	3HV/PHA (%)	*M_n_* × 10^5^ (g/mol)	*M_w_* × 10^5^ (g/mol)	*PDI*	*T_g_* (°C)	*T_c_* (°C)	*T_m_* (°C)
A	20/0	0.0	5.08	7.22	1.30	7.7	56.5	181.2
B	15/5	0.2	4.83	6.36	1.32	6.2	53.9	180.0
C	10/10	0.6	4.27	5.74	1.34	6.6	54.4	179.1
D	5/15	0.0	3.49	4.89	1.40	5.6	49.9	178.7
E	0/5	0.0	3.44	4.85	1.41	6.9	49.6	177.7
F	0/10	n.d.	n.d.	n.d.	n.d.	n.d.	n.d.	n.d.

n.d.: not determined.

### 2.2. Bioreactor Fermentation

**Figure 2:**
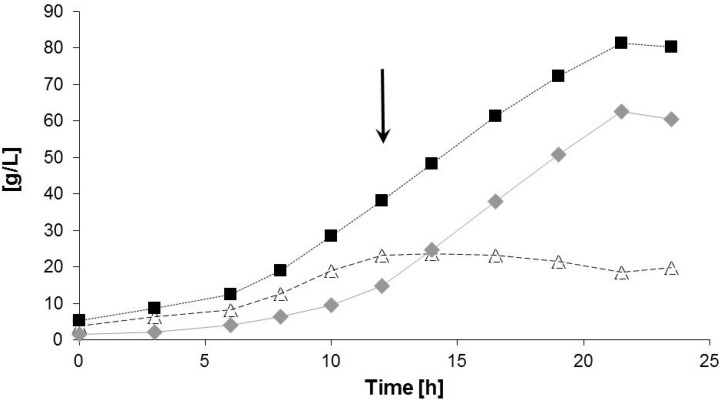
Pattern of product formation during the bioreactor fermentation: Cell dry mass (CDM, black squares), residual biomass (Xr, white triangles), and PHA (grey diamonds). The arrow indicates the stop of nitrogen (NH_4_^+^) supply to provoke PHA biosynthesis, and the start of LW-addition.

**Figure 3:**
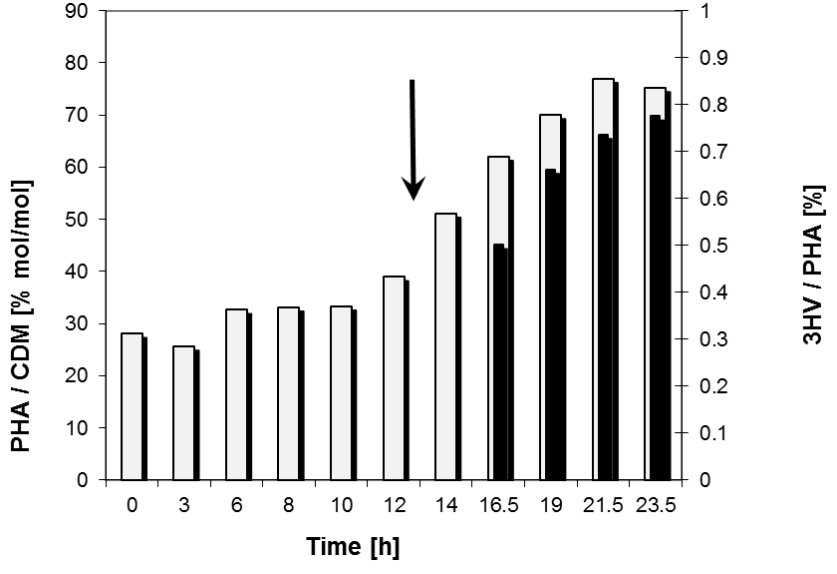
PHA content in cells (*grey bars*) and percentage of 3HV in the accumulated PHA (*black bars*) during the entire fermentation. The arrow indicates the stop of nitrogen (NH_4_^+^) supply and the start of LW-addition.

The results of the bioreactor fermentation are shown in Figure 2 and Figure 3 and Table 2, respectively. Figure 2 shows the time curves of CDM, residual biomass and PHA. The maximum specific growth rate (µ_max._) was calculated from the exponential phase of microbial growth (*t* = 6–12 h) and amounted to 0.20 1/h, which can be considered as fast growth for this strain [27,28]. The final concentration of catalytically active biomass amounted to 19.8 g/L; this value was reached after 12 h of cultivation. After this, the supply with nitrogen source (NH_4_^+^) was stopped in order to provoke the phase of PHA accumulation. From this time, the concentration of residual biomass remained constant, and the linear increase of CDM corresponds to the increase of the intracellular PHA content. It is well visible from Figure 2 that considerable amounts of PHA (around 30% in CDM) were already produced during the phase of balanced growth; this “partially growth associated PHA formation” [27] was observed from 0 h to 12 h. At the end of the experiment (*t* = 23.5 h), CDM and PHA were determined with 80.3 g/L and 60.5 g/L, respectively; this corresponds to a PHA content in biomass of 75.3%. The volumetric productivity for PHA amounted to a value of 2.84 g/(L h) for the entire production process; this is significantly higher than data obtained in similar laboratory production setups using *C. necator* on glucose plus complex growth additives (maximum values 0.65 g/(L h); see [28]).

At the end of the fermentation, the share of 3HV in PHA, caused by the addition of LW, amounted to 0.8% (mol/mol). Due to the fact that the addition of LW only started after 15 h of the cultivation, the cells accumulated PHB homopolyester before; after the sampling at *t* = 16.5 h, 3HV building blocks are evidenced in the polyester (Figure 3).

The most significant kinetic results of the process (max. specific growth rate µ_max._, max. specific productivity, volumetric productivity, maximum values for CDM and PHA concentration, maximum mass fraction of PHA in CDM, and molar 3HV fraction) and data for polymer characterization (molar mass distribution and thermal characterization) are collected in Table 2.

**Table 2:** Main results of the bioreactor fermentation: Kinetics and polymer characterization.

Data for Bioprocess	Time Period (h)	
Max. cell dry mass (CDM)	21.5	80.3 (g/L)
Max. PHA concentration	21.5	60.5 (g/L)
Max. PHA content in cell mass	21.5	77.0 (% (w/w))
Max. 3HV content in PHA	23.5	0.8 (% (w/w))
Volumetric productivity for PHA	0–23.5	2.84 (g/(L h))
Specific volumetric productivity	12–21.5	0.14 (g/(g h))
Maximum specific growth rate µ_max._	6–12	0.20 (1/h)
Data for Polymer Characterization		
Number average molar mass *M_n_* × 10^5^	23.5	3.52 (g/mol)
Weight average molar mass *M_w_* × 10^5^	23.5	5.39 (g/mol)
Polydispersity index (*ĐM* = *M_w_*/*M_n_*)	23.5	1.53
Melting point (*T_m_*)	23.5	176.3 (°C)
Melting enthalpy (δ*Hm*)	23.5	90.7 (J/g)
Degree of crystallnity	23.5	62.1 (%)
Cold crystallization (*T_c_*)	23.5	54.7 (°C)
Glass transition temperature (*T_g_*)	23.5	5.9 (°C)

## 3. Discussion

The high productivity for PHA obtained in the bioreactor experiment (2.84 g/(L h)) was enabled by the high concentration of residual, catalytically active biomass (about 20 g/L) together with the high intracellular PHA content (75.3%). This value is superior to PHA productivity by this strain reported for comparable fed-batch processes on industrial scale using hydrolyzed cane sugar as carbon source (1.44 g/(L h); see [29]). Concerning the specific volumetric productivity, the achieved value of 0.14 g/(g h) was considerably higher than comparable data reported before, classically below 0.10 [28].

Results from characterization of the polymer are in a similar range if compared to the polymer with the highest 3HV content (0.6% mol/mol) produced on shake flask scale. As a major outcome, the produced PHA was a poly(3HB-*co*-0.8%-3HV) copolyester with high molar mass (*M_w_* = 5.39 × 10^5^ g/mol; *M_n_* = 3.52 × 10^5^ g/mol) and low dispersity (*Đ_M_* = 1.53). This indicates a narrow molecular mass distribution was isolated from the cells. This molar mass was significantly higher than reported elsewhere for PHA industrially produced by this microbial species from cane sugar and cane sugar molasses [29]. *Đ_M_* for PHA produced by this strain are reported in a range of 3 [29]) to 4 [28]; hence, the results obtained by the work at hands were superior to literature data in terms of molar mass distribution. The melting temperature (176.3 °C) was slightly lower than reported for PHB homopolyester produced by *C. necator* on glucose using laboratory bioreactors (180 ± 1 °C; see [28]), and in a similar range as for PHB produced by the same strain on cane sugar (173.5 °C; see [29]).

Asbhy *et al.* used glycerol and levulinic acid as pure substrates to cultivate *Pseudomonas oleovorans*, biosynthesizing PHAs with much higher 3HV content [30]. Our results are largely in accordance with this observation. Namely, when the general liquefaction method was applied to cotton containing close to 100 wt % cellulose approx. 4% levulinic acid was obtained [30]. The much more complex and more “crude” spruce wood with a significantly lower content of cellulose (approx. 40%) can be expected to yield significantly lower levulinic acid contents given that levulinic acid is derived from cellulose through glucose and 5-hydroxymethylfurfural; the detailed analysis of our liquification products and elucidation of the involved reaction mechanisms will be the objectives of next studies. The obtained results are promising since spruce wood also contains components such as terpenoids and lignin that are known to inhibit microbial activity and PHA production. In order to obtain co-polyesters with sufficiently modified properties to increase their applicability, the content of non-3HB comonomer content does not need to be very high. We thus conclude that the results shown could be significantly improved by choosing a substrate with higher cellulose content and lower content of inhibiting compounds.

## 4. Experimental Section

### 4.1. Production of Liquified Wood (LW)

LW was prepared in a batch process according to [19] using a milestone laboratory microwave oven with sealed 100 mL PTFE-lined reactors. The wood used was spruce in form of dry sawdust. Each reactor was charged with 10 g sawdust, 30 g glycerol, 0.5 g *p*-toluenesulfonic acid, sealed and heated 2 min at 500 W microwave power and 5 min at 300 W. The temperature achieved was between 190 and 200 °C. After the reaction and partial cooling, the mixture was filtered and neutralized by 0.1 M (*aq.*) NaOH to yield a viscous dark liquid. During filtering, 7.4 wt % of the wood was removed as particulate residue. The process was adjusted to the intended use as bio-substrate by using glycerol and avoiding other glycols known to have inhibitory or toxic effects on bacteria and using an organic strong acid in place of mineral acids. The neutralization avoided ammonia in order not to introduce nitrogen into the substrate.

### 4.2. Production Strain

*Cupriavidus necator* DSM 545 (formerly known as *Wautersia eutropha*, *Ralstonia eutropha*, *Alcaligenes eutrophus*, or *Hydrogenomonas eutropha* [31]), bacteriologically a member of the Burkholderiaceae family [32], is a well-known producer of “simple” thermoplastic PHAs such as PHB or poly(3HB-*co*-3HV) [27,28,29] and less crystalline 4HB-containing copolyesters [33]. The strain was obtained as lyophilized culture from DSMZ culture collection (Braunschweig, Germany).

### 4.3. Cultivation Conditions and Shake Flask Experiment

#### 4.3.1. Strain Maintenance

Strain maintenance was done on agar slants (minimal medium according to Küng [34]) that were stored at 4 °C. Colonies were transferred to fresh slants in two-week intervals.

#### 4.3.2. Shake Flask Experiment

The shake flask experiment was designed as a two-stage process, separated into a growth phase and a nitrogen-limited PHA accumulation phase.

Inoculum preparation: Inoculum cultures were prepared by transferring single colonies from agar slants into baffled 300 mL shake flasks containing 100 mL of fresh medium composed as follows (per liter): KH_2_PO_4_, 5.0 g; (NH_4_)_2_SO_4_, 2.5 g; MgSO_4_·7H_2_O, 0.8 g; NaCl, 1.0 g; CaCl_2_·2H_2_O, 0.02 g; NH_4_Fe(III)Citrate, 0.05 g; traces elements solution SL6 (composition reported by [35]) 2.5 mL/L; glucose, 20 g (sole carbon source). The pH-value was adjusted to 7.0, media compounds were heat sterilized in an autoclave (20 minutes at 121 °C). The cultivation was performed at *T* = 30 °C for 16 h under continuous shaking on a rotary shaker.

Growth phase: 5 mL of inoculum culture prepared according to *Inoculum preparation* were transferred into baffled 1 L shake flasks containing 300 mL of fresh medium and cultivated for 16 h on a rotary shaker (130 rpm) under the same conditions.

Accumulation phase: After the growth phase cultivation described above, the cells were separated from the supernatant by centrifugation (Sorvall RC-5B Refrigerated Superspeed centrifuge, 14,500× *g*, 10 min) (Fisher Scientific GmbH, Vienna, Austria), re-suspended in sterile NaCl solution (0.9%) to remove residues of the nutritional medium, centrifuged again and finally re-suspended in a nitrogen-limited medium in order to provoke PHA accumulation. The composition of this nitrogen-limited medium was the same as for the first stage of cultivation (growth phase) without addition of the nitrogen source (NH_4_)_2_SO_4_. As a carbon source, different mixtures of glucose and LW were added as reported in Table 3. The cultivations were accomplished by continuous shaking for 24 h on a rotary shaker at 30 °C (130 rpm) and initial pH-value of 7.0. Samples of the culture broth were taken at the beginning and at the end (24 h) for further analyses. All cultivation set-ups were performed in duplicate.

**Table 3:** Supplied quantities of the carbon sources glucose (GLU) and liquefied wood (LW): *upriavidus necator* DSM 545 under PHA-accumulating conditions on shake flask scale.

Experimental Set-Up	GLU (g/L)	(LW) (g/L) *
A	20	0
B	15	5
C	10	10
D	5	15
E	0	5
F	0	10

* Values refer to dry mass of LW.

### 4.4. Bioreactor Experiment

For the bioreactor fermentation, a 7.5 L laboratory Labfors 3 stirred tank reactor (Infors, Basel, Switzerland), equipped with two axial-propeller stirrers, was used. Temperature (30 °C), pH-value (7.0 ± 0.2; probe *Easyferm PLUS K8*, Hamilton, ON, Canada) and the oxygen partial pressure pO_2_ (probe *Oxyferm FDA*, Hamilton, ON, Canada) were controlled and sustained automatically. The concentration of dissolved oxygen was controlled by adjustment of the agitation speed of the stirrer and kept at around 40% of the saturation concentration of oxygen in water during the phase of balanced growth, and 20% of the saturation concentration during the phase of predominant PHA production. The oxygen supply was performed by aeration at 150 L/h through an absolute filter (Sartorius, Midisart 2000, Göttingen, Germany). The bioreactor was filled with 4 L of medium and inoculated with 1 L of a dense pre-culture from the late exponential phase (optical density at λ = 420 nm about 20, initial OD around 0.1) that was cultivated in baffled shake flasks for 16 h. The cultivation medium was composed as follows (per liter): KH_2_PO_4_, 5.0 g; (NH_4_)_2_SO_4_, 2.5 g; MgSO_4_·7H_2_O, 0.8 g; NaCl, 1.0 g; CaCl_2_·2H_2_O, 0.02 g; NH_4_Fe(III)Citrate, 0.05 g; traces elements solution SL6 2.5 mL/L; Glucose, 20 g; LW was added drop-wise during the PHA accumulation phase.

Nitrogen source (NH_4_^+^) was selected as growth limiting factor. The nitrogen supply was accomplished by using NH_4_OH solution (*aq.*, 25% w/w) for correction of the pH-value that drops in parallel to biomass growth. Hence, during growth phase, both nitrogen supply and correction of pH value were accomplished in parallel by NH_4_OH supply. After 12 h of growth, the supply of nitrogen was stopped by exchanging NH_4_OH solution by *aq.* NaOH solution (10%) in order to provoke nitrogen limited conditions, thus resulting in the switch from growth phase to PHA accumulation phase. After a duration of 15 h, LW was added drop-wise by six equal feed pulses until the end of the fermentation at *t* = 24 h; this was done in order to minimize toxic effects of LW if the entire amounts is added at once as expected by the results from shake flasks. Until the end of the process, a total amount of 10 g/L (calculated for LW dry mass) was added. During the entire process, glucose was added by a total of 13 feed pulses of an *aq.* 50% w/w solution according to HPLC measurements (data not shown; a total of 211 g/L glucose was added to the fermentation medium (start concetration plus re-feeds)) that were accomplished at regular times after each sampling.

### 4.5. Determination of Cell Dry Mass (CDM), Glucose and Ammonia

Five mL of fermentation broth was centrifuged in pre–weighed glass tubes. The supernatant was used for substrate analysis (NH_4_^+^, glucose), the remaining biomass pellet was frozen and lyophilized for 24 h (freeze-dryer Christ Alpha 1-4 B) (Martin Christ Gefriertrocknungsanlagen GmbH, Osterode am Harz, Germany). Gravimetric difference against empty tubes was calculated after weighing; the pellets were later used for determination of the PHA content. The determinations were done in duplicate. Glucose and ammonia was monitored according to [28].

### 4.6. PHA Analysis

Intracellular PHA in lyophilized biomass pellets as obtained by each sampling as described before (prior paragraph *Determination of CDM*) was transesterificated via acidic methanolysis by a method adapted from Braunegg *et al.* [36] and detailed in [37]. As internal standard, hexanoic acid was used. The analysis was carried out using a HP 5890 Series II gas chromatograph (30 m HP5 column, protected by a 5 m HP 1 capillary pre-column) (Hewlett-Packard Gesellschaft mbH, Vienna, Austria). The volatile transesterification products (methyl esters of the PHA building blocks 3HB and 3HV) were registered by a flame ionization detector (FID) (Hewlett-Packard Gesellschaft mbH, Vienna, Austria), the carrier gas helium was used with a split- ratio of 1:10. As reference, pure poly(3HB-*co*-19.1 mol-%-3HV) standards (ICI, London, UK) were used. The PHA content (% (w/w)) was defined as the percentage of the ratio of PHA concentration to dry cell mass (CDM). Residual biomass (Xr) (g/L) was defined as the difference of CDM (g/L) and PHA content (g/L).

### 4.7. PHA Recovery

The cells cultivated in shake flasks or in the bioreactor were *in situ* pasteurized (80 °C, 30 min), harvested by centrifugation (20 min at 4 °C and 6000 rpm in Sorvall^®^ RC-5B Refrigerated Superspeed centrifuge, DuPont Instruments, Newtown, CT, USA), frozen and lyophilized for 24 h. After degreasing the biomass by overnight Soxhlet extraction with the PHA-non solvent ethanol and subsequent drying at room temperature, PHA was Soxhlet-extracted overnight with the PHA solvent CHCl_3_. The volume of the obtained solution of PHA in CHCl_3_ was reduced to about 10% on a rotary evaporator (Büchi Rotavapor RE111, Flawil, Switzerland). From the remaining viscous solution, the polymer was precipitated by dropping the solution into the tenfold amount of iced ethanol under continuous stirring, recovered via vacuum-assisted filtration, and finally air dried at room temperature. The purity of the extracted materials as well as the completeness of the extraction was determined by GC analysis of the isolated PHA and the remaining biomass.

### 4.8. Determination of Melting Temperature, Glass Transition Temperature and Crystallinity

Thermal analysis characterization for determination of melting temperature (*T_m_*), glass transition temperature (*T_g_*), cold crystallization temperature (*T_c_*) and melting enthalpy (δ*H_m_*) was performed on a Mettler Toledo DSC 1 STAR^e^ System (Mettler-Toledo GmbH, Vienna, Austria) and associated software.

DSC samples of approximately 5 mg were weighed in 40 µL aluminium pans with an empty pan as reference. Measurements were carried out at a nitrogen flow rate of 20 mL/min according to the following protocol: first, second and third heating from −30 to 200 °C at 10 °C/min; first cooling (quenching after the first heating) from 200 to −30 °C at 100 °C/min and the second cooling from 200 to −30 °C at 10 °C /min. All thermodynamic parameters are reported from the second heating scan.

The degree of crystallinity (*X_c_*) was determined by considering the value of the melting enthalpy of 146 J/g for the 100% crystalline PHB [38].

### 4.9. PHA Molar Mass Characterization

Molar mass data were obtained from measurements on a Agilent 1100 series system equipped with a refractive index detector at 30 °C (Agilent 1100 Series, Agilent Technologies Österreich GmbH, Vienna, Austria) equipped with a SDV linear XL, 5 µm pre-column/column (300 × 7.5 mm, Polymer Standards Service, Mainz, Germany). CHCl_3_ was used as solvent (flow rate 1.0 mL/min, pressure 47 bar) and monodisperse polystyrene standards were used for calibration. Injected sample volume and concentration were 100 µL and 0.0025 g/mL, respectively. Prior to measurement the samples were dissolved for 1 h at 60 °C (atmospheric pressure, reflux condenser) in acid-free chloroform.

## 5. Conclusions

The proof of concept to use LW as 3HV-precursor was successfully demonstrated. Further activities have to overcome negative side effects of LW on the cultivation of *C. necator* by removal of toxic compounds via adsorption on charcoal, and by the identification of these compounds and their subsequent enzymatic inactivation. The exact composition of the liquefaction “cocktail” for using the applied substrate (saw dust of spruce wood), and details about the reaction mechanism have to be elaborated. In addition, continuous dual growth-limited biotechnological cultivation systems could be applied for the fermentation process, similar to PHA biosynthesis on other rather toxic substrates [39]. This way, higher amounts of LW, hence higher quantities of odd-numbered, 3HV-related compounds can be supplied to the cells to increase the 3HV content in PHA for additionally improving the material properties.
